# Caffeine as a promotor of sexual development in sterile Queensland fruit fly males

**DOI:** 10.1038/s41598-020-71671-x

**Published:** 2020-09-08

**Authors:** Saleh Mohammad Adnan, Iffat Farhana, Soo Jean Park, Polychronis Rempoulakis, Phillip W. Taylor

**Affiliations:** 1 Applied BioSciences, Macquarie University, Sydney, NSW 2109 Australia; 2 Biosecurity and Food Safety, NSW Department of Primary Industries, Gosford, Australia; 3 Department of Molecular Sciences, Macquarie University, Sydney, NSW 2109 Australia

**Keywords:** Entomology, Reproductive biology

## Abstract

Sterile insect technique (SIT) is an environmentally benign pest management technique that involves releasing millions of sterile insects to suppress reproduction of pest populations. Many fruit flies, including Queensland fruit fly (*Bactrocera tryoni* Froggatt, ‘Q-fly’), have long adult maturation periods such that pre-maturation mortality can greatly reduce abundance of sexually active sterile males and impede SIT efficacy. Q-fly is the most difficult and costly challenge to market access for Australia’s horticulture industries, and has been targeted for intensive use of SIT program. We here demonstrate potential of pre-release caffeine supplements as a novel means to accelerate sexual maturation in male Q-fly. In mating trials, analytical caffeine was very effective at accelerating sexual maturation, while no positive effects of caffeine-containing instant coffee or guarana supplements were detected. In parallel, development of testes and ejaculatory apodemes was accelerated in males provided analytical caffeine but not instant coffee or guarana. High doses of guarana and instant coffee reduced longevity while even the highest doses of analytical caffeine did not affect longevity. Pre-release caffeine supplements promote sexual maturation in Q-flies, and similar benefits are expected in other fruit flies having long adult maturation periods.

## Introduction

Tephritid fruit flies are amongst the world's most economically significant pests of fruit production^1,2^. In Australia, the Queensland fruit fly, *Bactrocera tryoni* (Froggatt), or ‘Q-fly’, presents a costly challenge to Australia’s $13.5 billion horticulture industry, affecting a vast diversity of commercial and non-commercial crops^3–5^. Organophosphate insecticides have been the most common solution for decades, but their use is now greatly restricted due to environmental and human health concerns. Alternative control measures are now a high priority. The Sterile Insect Technique (SIT) is a sustainable technology that is growing rapidly in favour. SIT involves releasing millions of sterile insects that reduce the reproductive capacity of pest populations by inducing reproductive failure in females^6,7^. SIT has been used globally to combat some of the most damaging fruit fly species, including the Mediterranean fruit fly, or medfly *Ceratitis capitata* (Wiedemann)^8^, the Melon fly *Zeugodacus cucurbitae* (Coquillett)^9,10^, the Oriental fruit fly *Bactrocera dorsalis* (Hendel)^11^ and the Mexican fruit fly *Anastrepha ludens* (Loew)^12^. While SIT has proven effective for numerous species, there is still substantial scope to increase efficacy and cost effectiveness in most programs.

Fruit flies commonly have long adult maturation phases and high mortality rates in the field, such that a quite small proportion of the released flies might survive to mature and contribute to SIT^13–16^. Most species are anautogenous, needing to acquire nutritional resources, especially protein, as adults to complete reproductive development^17^. Yeast hydrolysate (YH) mixed with sugar is a standard adult diet used to maintain fruit fly colonies, providing a rich source of amino acids, micronutrients and carbohydrates^18^ that is effective at sustaining reproductive development^19–21^. When provided for even a 1–2 day pre-release period in SIT programs, YH and sugar effectively sustain development of male Q-flies over the following days^22,23^, increasing the prevalence of mature sterile flies in the field^24^.

While providing sterile Q-flies with nutritional resources during pre-release holding periods is an effective means of hastening development, additional treatments that can further reduce the delay between release and maturity remain of particular interest. Dietary or topical application of methoprene, a juvenile hormone analogue, provides one prominent avenue, having been found to substantially accelerate adult development in Q-fly^25–28^, as well as in *Z. cucurbitae*^29,30^, *A*. *fraterculus*^31–34^, *A. ludens*^35–37^, and *A. suspensa*^38,39^. Other pre-release treatments have been explored for their effects on sexual performance of already mature fruit flies, especially plant semiocmicals such as methyl eugenol to which males of some species are attracted and from which they gain substantial sexual benefits^40–47^. While Q-flies are not attracted to methyl-eugenol, they are attracted to raspberry ketone. Dietary access to cuelure, an analogue of raspberry ketone, has been found to function as a potent simulant in sexually mature male Q-flies, significantly increasing expression of energetic pathways and significantly increasing sexual performance for 2–3 days^48^. This effect has been colloquially termed ‘The Red Bull Effect’^48^. Although immature males are not attracted to raspberry ketone, Akter et al.^49^ has found that raspberry ketone mixed in the diet of immature male Q-flies yields acceleration in sexual maturation that is a close match to the effects of methoprene^27,28^. The finding that a compound known to be effective as a stimulant in mature adult Q-flies also accelerates development of immature adult Q-flies raises the possibility that other stimulants might also accelerate development of fruit flies released in SIT programs.

Caffeine is a potent stimulant, affecting diverse taxa including insects^50–56^. Several studies that point to possible effects of caffeine in tephritid fruit flies, including effects on mating performance. Arita and Kaneshiro^57^ compared sexual competitiveness of male *C. capitata* from two populations, one that emerged from Jerusalem cherries (*Solanum pseudocapsicum* L.) and one that emerged from Arabian coffee (*Coffea arabica* L.). Despite being smaller, the male flies emerging from coffee sexually outcompeted the male flies emerging from Jerusalem cherries, regardless of which population the females came from. One possible explanation is that exposure to caffiene during the larval stage carried over to the adult stage, then either accelerated development or increased sexual effort. In a mating competitiveness study, laboratory *C. capitata* provided ad libitum access to adult diet supplemented with guarana powder, which contains caffiene, sexually outperformed untreated laboratory and wild flies^58^. It remains unclear, however, whether these results are attributable to the caffiene or other compounds in the supplements, and whether the effects were through accleration of development or through elevated levels of sexual effort.

In the present study we investigate whether sexual maturation of male Q-flies is accelerated by caffeine supplements from three different sources—analytical caffeine, guarana powder, and instant coffee incorporated into the adult diet for two days following adult emergence. Because such supplements could increase mating activity without promoting development of reproductive organs^22^, we assess the effects of treatment and age on both mating success and the development of reproductive organs as separate measures of sexual maturation.

## Results

### Longevity

**Figure 1:**
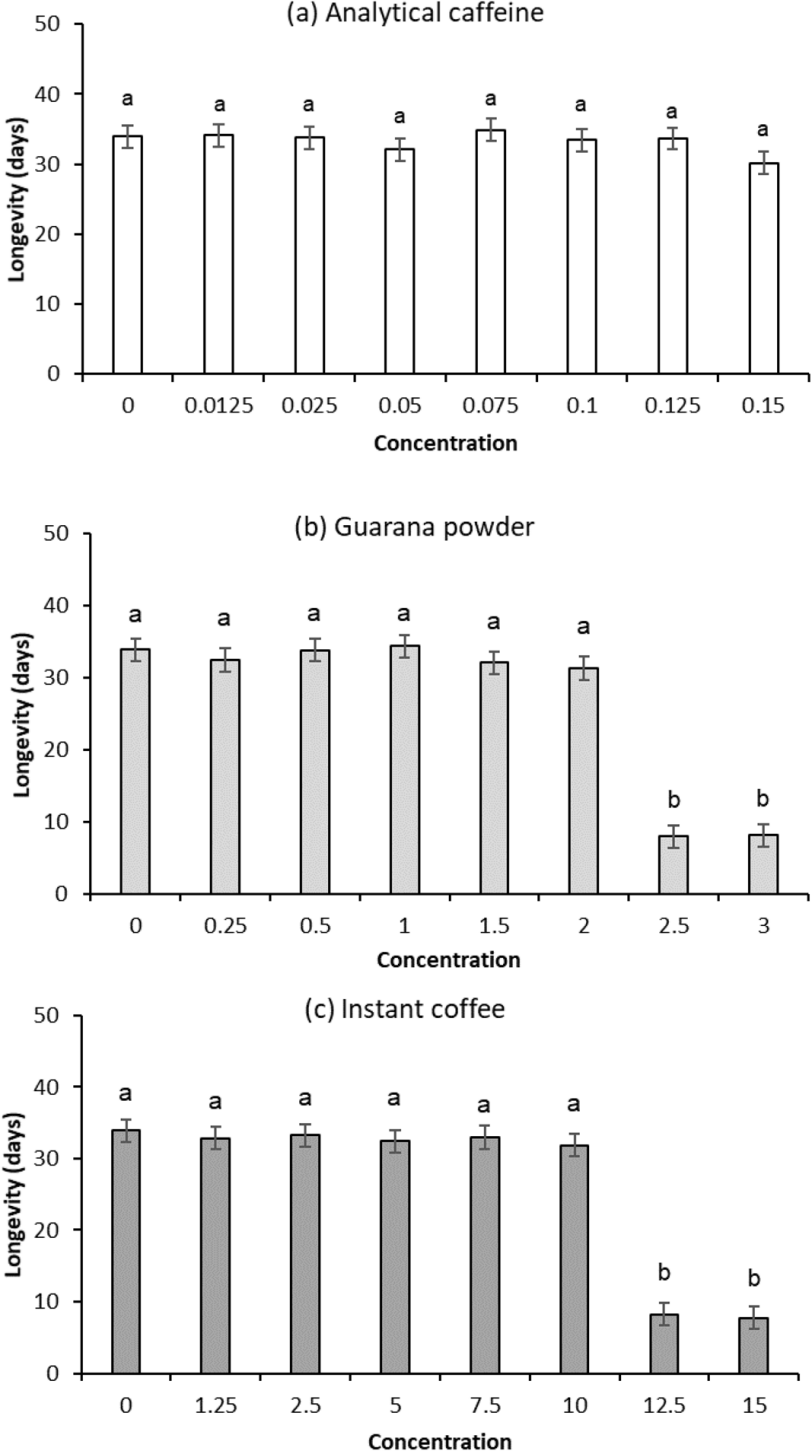
Effects of dose (%) of each supplement (Analytical caffeine, Guarana powder, Instant Coffee and Control) on male Q-fly longevity. Means with different letter from control are significantly different from control (Dunnett’s test, *P* < 0.05).

Analytical caffeine had no significant effect on Q-fly longevity at any tested concentration (F_7,71_ = 0.5, P = 0.85; Fig. 1a), whereas the highest two doses of both guarana powder (F_7,71_ = 97.5, P < 0.001; Fig. 1b) and instant coffee (F_7,71_ = 78.7, P < 0.001; Fig. 1c) significantly reduced longevity.

### Sexual performance

#### Mating probability

**Figure 2:**
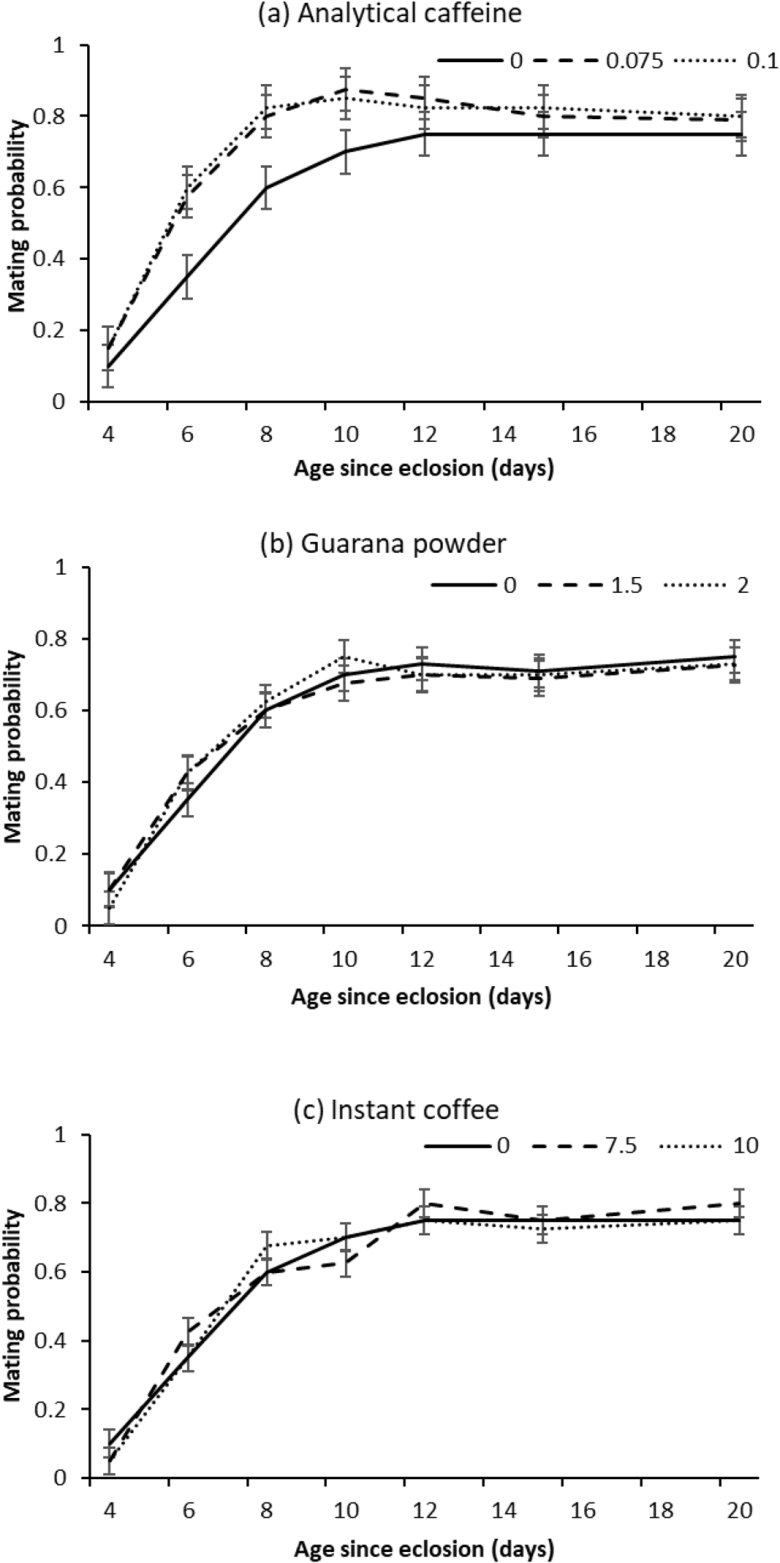
Relationship between age and proportion of male Q-flies mating after being fed a diet of sugar and yeast hydrolysate combined with two doses (%) of each supplement (Analytical caffeine, Guarana powder, Instant coffee and Control) for 2 days after emergence and then sugar only.

For all treatments, age had significant effect on mating probability (Table 1) as the proportion of flies mating increased over the first 8 days of adulthood, and then plateaued (Fig. 2). In addition to the effects of age, mating probability significantly varied with analytical caffeine treatment (Table 1). Flies provided both doses of analytical caffeine had significantly greater mating probability than the control (low dose log odds = 0.71 ± 0.2, P = 0.0011; high dose log odds = 0.66 ± 0.2, P = 0.0022; Fig. 2a). No effects were detected for either guarana powder or instant coffee (Table 1, Fig. 2b,c).

**Table 1:** GLM analysis testing fixed effects of age (4, 6, 8, 10, 12, 15, and 20 days post emergence) and treatments (Analytical caffeine, Guarana powder, Instant coffee and Control) on male Q-fly mating probability.

Treatment	Variable	*d.f*	*χ *^2^	*P*
Analytical caffeine	Dose	2	12.000	0.0025
	Age	6	202.000	< 0.001
	Dose × age	12	5.200	0.95
Guarana powder	Dose	2	0.100	0.94
	Age	6	188.500	< 0.001
	Dose × age	12	3.500	0.99
Instant coffee	Dose	2	0.400	0.84
	Age	6	216.700	< 0.001
	Dose × age	12	3.100	1.00

#### Mating latency

**Figure 3:**
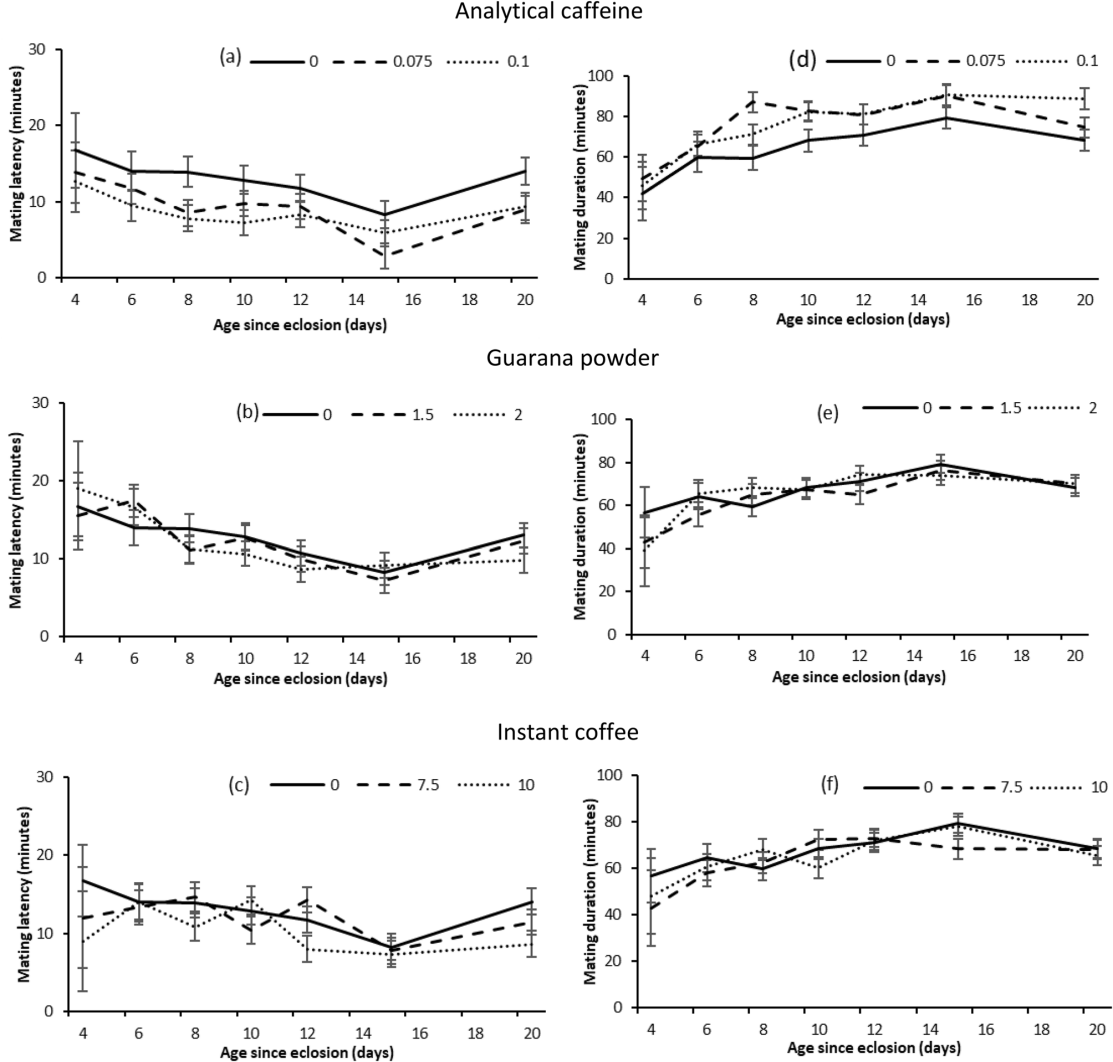
Mating latency (mean ± SE) and mating duration (mean ± SE) obtained in mating trials at different ages by male Q-flies provided a diet of sugar and yeast hydrolysate combined with two doses (%) of each supplement (Analytical caffeine, Guarana powder, Instant coffee and Control) for 2 days after emergence and then sugar only.

For all treatments, mating latency varied significantly with age (Table 2). Latency to mate decreased with age until 15 days (Fig. 3a–c). As with mating probability, analytical caffeine treatment had significant effect on mating latency (Table 2). Flies that received both doses of analytical caffeine had significantly shorter mating latency than untreated flies (low dose $\Delta \beta \hspace{0.17em}$= 0.45 ± 0.12, P < 0.001; high dose $\Delta \beta$ = 0.5 ± 0.12, P < 0.001; Fig. 3a). However, flies provided guarana powder and instant coffee had mating latency that was not significantly different from untreated flies (Table 2, Fig. 3b).

**Table 2:** Linear model analysis testing fixed effects of age (4, 6, 8, 10, 12, 15, and 20 days post emergence) and treatments (Analytical caffeine, Guarana powder, Instant coffee and Control) on male Q-fly mating latency.

Treatment	Variable	*d.f*	*F*	*P*
Analytical caffeine	Dose	2,523	9.600	< 0.001
	Age	6,523	4.000	< 0.001
	Dose × age	12,523	0.300	0.99
Guarana powder	Dose	2,448	1.500	0.23
	Age	6,448	5.200	< 0.001
	Dose × age	12,448	0.500	0.90
Instant coffee	Dose	2,461	2.400	0.09
	Age	6,461	3.000	0.0078
	Dose × age	12,461	1.000	0.47

#### Mating duration

Age had significant effect on mating duration for all treatments (Table 3). Mating duration increased with age until 15 days (Fig. 3d–f). Mating duration also varied significantly with analytical caffeine treatment (Table 3). Flies that received both doses of analytical caffeine had significantly longer mating duration than untreated flies (low dose $\Delta \beta$ = 0.69 ± 0.17, P < 0.001; high dose $\Delta \beta$ = 0.6 ± 0.17, P < 0.001; Fig. 3d). However, mating durations of flies provided guarana powder or instant coffee were not significantly different from those of control flies (Table 3; Fig. 3e,f).

**Table 3:** Linear model analysis testing fixed effects of age (4, 6, 8, 10, 12, 15, and 20 days post emergence) and treatments (Analytical caffeine, Guarana powder, Instant coffee and Control) on male Q-fly mating duration.

Treatment	Variable	*d.f*	*F*	*P*
Analytical caffeine	Dose	2,523	8.100	< 0.001
	Age	6,523	8.600	< 0.001
	Dose × age	12,523	1.400	0.18
Guarana powder	Dose	2,448	1.000	0.37
	Age	6,448	4.400	< 0.001
	Dose × age	12,448	0.600	0.80
Instant coffee	Dose	2,461	0.300	0.71
	Age	6,461	3.800	< 0.001
	Dose × age	12,461	1.000	0.49

### Reproductive organ development

#### Ejaculatory apodeme

**Figure 4:**
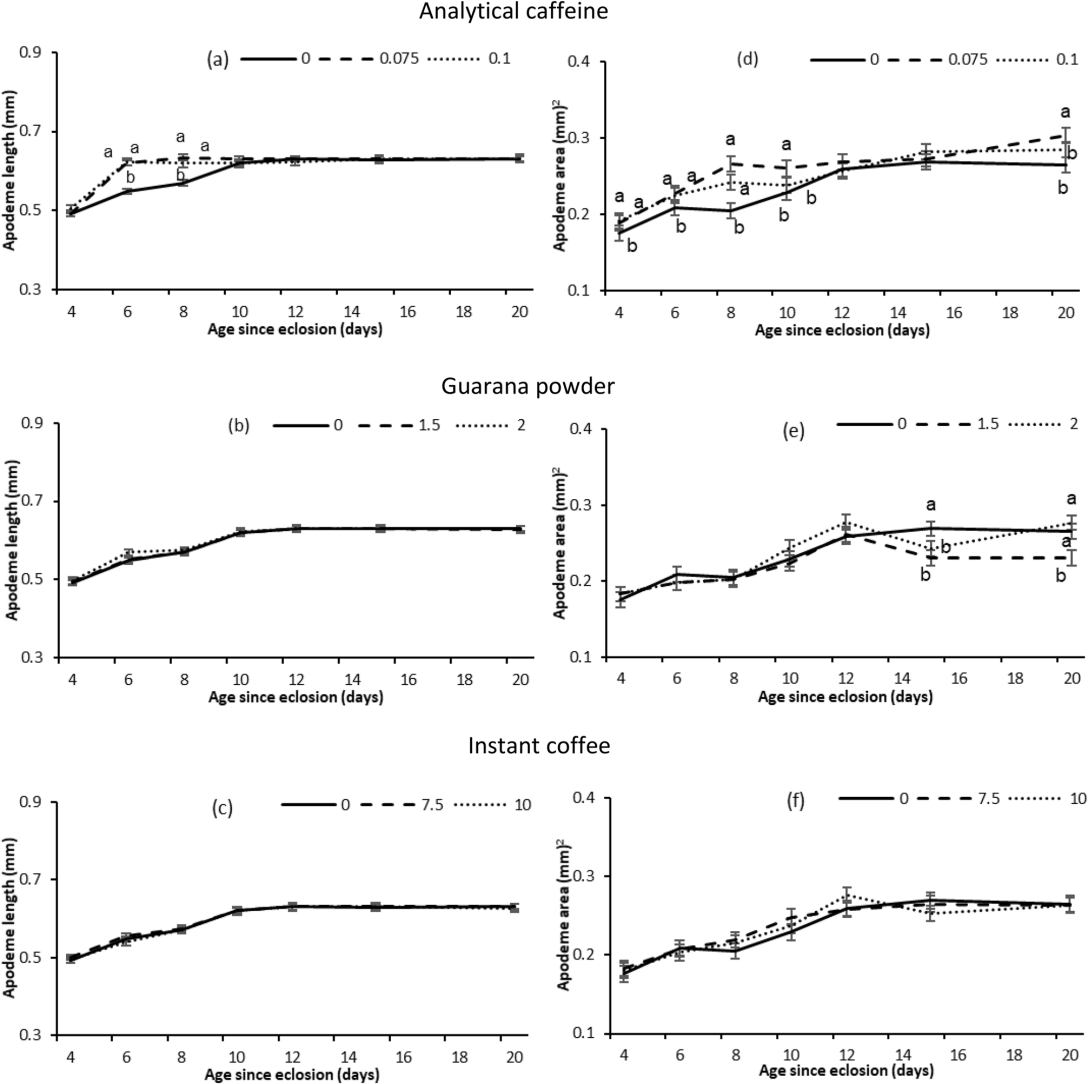
Ejaculatory apodeme length (mean ± SE) and apodeme area (mean ± SE) obtained at different ages by male Q-flies provided a diet of sugar and yeast hydrolysate combined with two doses (%) of each supplement (Analytical caffeine, Guarana powder, Instant coffee and Control) for 2 days after emergence and then sugar only. Means separated by different letters indicates significant differences between treatment and control at particular ages (Dunnett’s test, *P* < 0.05).

For flies provided analytical caffeine, ejaculatory apodeme length and area varied significantly with age and dose, with a significant age × dose interaction (Table 4) (Fig. 4). Ejaculatory apodeme length and area increased with age, but this increase was much steeper at young ages for flies that were provided the two doses of analytical caffeine compared with control flies (Fig. 4a,d). For flies provided guarana powder, ejaculatory apodeme length increased significantly with age but was not affected by the supplements (Table 4), while ejaculatory apodeme area varied with a significant age × dose interaction (Table 4). Flies that were provided guarana powder had reduced apodeme area at 15 and 20 days of age compared to control flies (Fig. 4e). For flies provided instant coffee ejaculatory apodeme length and area increased significantly with age but was not affected by the supplements (Table 4) (Fig. 4c,f).

**Table 4:** GLM analysis testing fixed effects of age (4, 6, 8, 10, 12, 15, and 20 days post emergence) and treatments (Analytical caffeine, Guarana powder, Instant coffee and Control) on male Q-fly ejaculatory apodeme development (length and area).

Response	Treatment	Variable	*d.f*	*F*	*P*
Length	Analytical caffeine	Dose	2,398	21	< 0.001
		Age	6,398	156	< 0.001
		Dose × age	12,398	6.5	< 0.001
	Guarana powder	Dose	2,398	1.2	0.30
		Age	6,398	164.7	< 0.001
		Dose × age	12,398	0.3	0.98
	Instant coffee	Dose	2,398	0.4	0.69
		Age	6,398	163.1	< 0.001
		Dose × age	12,398	0.1	1.00
Area	Analytical caffeine	Dose	2,398	34.6	< 0.001
		Age	6,398	106.9	< 0.001
		Dose × age	12,398	3.4	< 0.001
	Guarana powder	Dose	2,398	8	< 0.001
		Age	6,398	86.3	< 0.001
		Dose × age	12,398	3.3	< 0.001
	Instant coffee	Dose	2,398	1.6	0.21
		Age	6,398	104.5	< 0.001
		Dose × age	12,398	1.3	0.23

#### Testes

**Figure 5:**
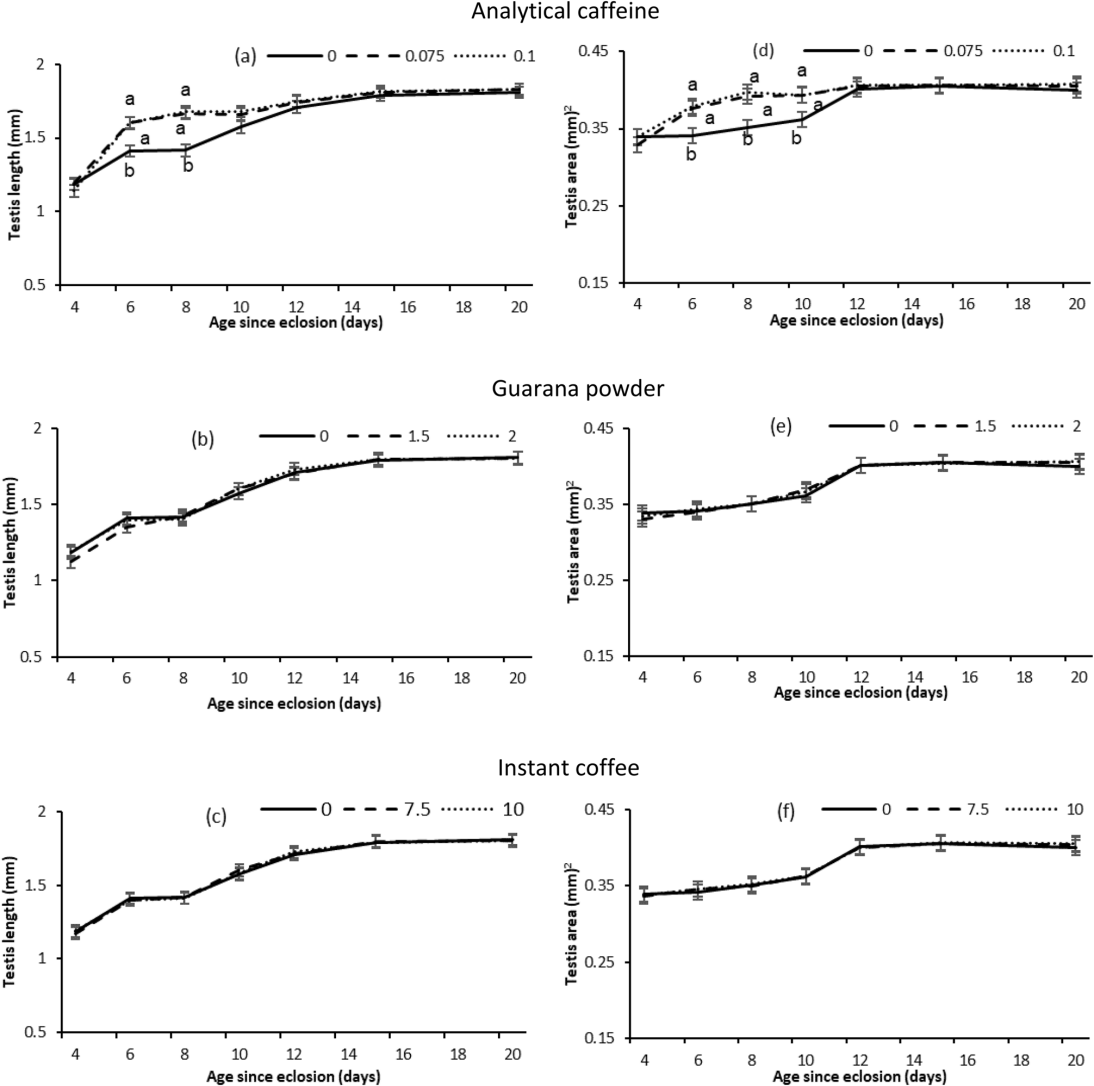
Testis length (mean ± SE) and testis area (mean ± SE) obtained at different ages by male Q-flies provided a diet of sugar and yeast hydrolysate combined with two doses (%) of each supplement (Analytical caffeine, Guarana powder, Instant coffee and Control) for 2 days after emergence and then sugar only. Means separated by different letters indicates significant differences between treatment and control at particular ages (Dunnett’s test, *P* < 0.05).

For flies provided analytical caffeine, testis length and area varied significantly with age and dose, with a significant age × dose interaction (Table 5; Fig. 5). Testis length and area increased with age, but this increase was much steeper at young ages for flies that were provided the two doses of analytical caffeine compared with control flies (Fig. 5a,d). For flies provided guarana powder and instant coffee, testis length and area increased significantly with age but was not affected by the supplements (Table 5; Fig. 5b,c,e,f).

**Table 5:** GLM analysis testing fixed effects of age (4, 6, 8, 10, 12, 15, and 20 days post emergence) and treatments (Analytical caffeine, Guarana powder, Instant coffee and Control) on male Q-fly Testis development (length and area).

Response	Treatment	Variable	*d.f*	*F*	*P*
Length	Analytical caffeine	Dose	2,398	12.2	< 0.001
		Age	6,398	123.4	< 0.001
		Dose × age	12,398	2.9	< 0.001
	Guarana powder	Dose	2,398	0.6	0.58
		Age	6,398	125.4	< 0.001
		Dose × age	12,398	0.4	0.97
	Instant coffee	Dose	2,398	0	0.99
		Age	6,398	131.2	< 0.001
		Dose × age	12,398	0.1	1.00
Area	Analytical caffeine	Dose	2,398	13	< 0.001
		Age	6,398	36.6	< 0.001
		Dose × age	12,398	2.4	0.0058
	Guarana powder	Dose	2,398	0	0.97
		Age	6,398	43.4	< 0.001
		Dose × age	12,398	0.1	1.00
	Instant coffee	Dose	2,398	0.1	0.89
		Age	6,398	47	< 0.001
		Dose × age	12,398	0.1	1.00

## Discussion

Dietary supplements of analytical caffeine significantly increased mating propensity and accelerated reproductive organ development in male Q-flies. These effects were closely comparable to effects of methoprene^25–27^ and raspberry ketone^49^ supplements incorporated into the adult diet for two days following emergence. Despite providing equivalent concentrations of caffeine in guarana powder and instant coffee, these supplements had little effect on mating behaviour or development.

Being a metabolic enhancer caffeine likely activates metabolic pathways in immature male Q-flies, and by this mechanism accelerates reproductive development. Caffeine is the most popular psychoactive drug in the world owing to its stimulatory properties^50,55^. In addition to the familiar effects on humans, caffeine also has potent effects on invertebrates such as increased spontaneous neural activity and increased locomotor activity^52,59^. While in invertebrates caffeine influences energy reserves and metabolic activity^60^, in vertebrates caffeine mostly acts on the central nervous system^61^. Sequentially it mobilizes intracellular calcium, inhibits specific phosphodiesterase, and most importantly it induces differential responses on the action of the methylxanthine on serotonin neurons. These responses are found to translate into altered locomotion, learning, memory, performance and coordination^52^. Furthermore, in vertebrates caffeine promotes energy metabolism in the brain and decreases cerebral blood flow, generating a relative brain hypo perfusion^61^. However, in *Drosophila* Nall et al.^53^ postulated that dopamine mediates the wake-promoting effect of caffeine, and that caffeine likely acts pre-synaptically to increase dopamine signalling. A cluster of neurons, the paired anterior medial (PAM) cluster of dopaminergic neurons exhibits increased activity as a result of caffeine administration^53^. Dopamine has been reported to increase behavioural activity (locomotion, sexual behaviour, and pheromone production or secretion) in *Drosophila melanogaster*^62–66^, to mediate aggressive encounter, flying and fighting ability in crickets^67,68^, to increase motor response in worker bees^69,70^ and queen bees^71,72^ and to enhance escape behaviour in cockroaches^73,74^. Therefore, accelerated sexual maturation in caffeine-supplemented Q-fly might be related to increased dopamine signalling. Further studies are required to address the changes in biogenic amines following caffeine treatment.

In addition to increased mating propensity, analytical caffeine-supplemented Q-fly males had shorter mating latency. It might be that shorter mating latency is simply a behavioural expression of greater sexual vigour and courtship effort. Shorter latency to initiate mating displayed by analytical caffeine-supplemented males corresponds with similar effect associated with methoprene supplements in the Q-fly^26^ and also in the melon fly *Bactrocera cucurbitae*^30^. Additionally, analytical caffeine-supplemented flies had longer mating duration, which also resembles the effect of dietary methoprene supplements^26,27,30^. The implications of longer mating duration for SIT is not straightforward. While there is little evidence of links between between mating duration and quantity of sperm storage^20,75–77^, postcopulatory success might still be promoted by longer copulation. Longer copulations by sterile males can induce higher levels of infertility in females that have previously mated with a fertile male or later remate with a fertile male, which is positive post-mating outcome for SIT^76^.

The increase in mating propensity of analytical caffeine-supplemented flies corresponded with accelerated development of the reproductive organs (ejaculatory apodeme and testes). This pattern confirms that the effects of analytical caffeine are not solely behavioural, but that the expression of mating behaviour aligns with development of the reproductive system. This is important, because if analytical caffeine only affected mating behaviour without also affecting morphological development then the matings of young caffeine treated males might be ineffective. While there is still a need to directly assess remating tendency of females mated by analytical caffeine-supplemented males, the alignment of age-dependent mating propensity with age-dependent development of reproductive organs is encouraging. Those treatments that did not result in increased mating propensity also did not result in accelerated development of reproductive organs, and this further substantiates the links between development of reproductive organs and expression of mating behaviour.

Regardless of dose, analytical caffeine did not affect the longevity of Q-flies. On the other hand, the highest two doses of guarana powder and instant coffee resulted in substantially reduced longevity. Caffeine is the most known component of roasted coffee beans, varying between 0.8 and 4.0% (w/w) depending on variety^78^. However, coffee beans also contain a large diversity of compounds, including carbohydrates (38.0–41.5%), lipids (11–17.0%), protein (10.0%), trigonelline, niacin (1.0%), aliphatic acids (2.4–2.5%), chlorogenic acids (2.7–3.1%), minerals (4.5–4.7%), melanoidinsc/brown compounds (23%)^78–80^. In addition to a high percentage (2.5–6%) of caffeine (1,3,7-tri-methylxanthine)^81^, guarana seeds also contain starch (60.88%), tannin (9.6%), protein (8.56%), soluble sugar (7.97%), reducing sugars (4.89%), as well as smaller proportions of the purine alkaloids theobromine (3,7-dimethylxanthine) and theophylline (1,3-dimethylxanthine). Guarana seeds also contain a high concentration of polyphenols, particularly proanthocyanidins with a higher prevalence of catechins and epicatechins^82–85^. Some of the diverse compounds in guarana powder and instant coffee might be toxic when applied at higher tested doses, or inhibit feeding, which would explain the reduction in Q-fly longevity. Despite having similar caffeine content, guarana powder and instant coffee did not affect sexual development and this may reflect sub-lethal effects of constituents other than caffeine. Overall, together with the acceleration of reproductive development, the absence of negative effects of analytical caffeine supplements on longevity are promising for use as a potential pre-release supplement for Q-fly SIT.

## Conclusion

Dietary analytical caffeine supplements provided in the first two days post-eclosion strongly accelerate Q-fly reproductive development without effects on longevity, and have potential value as pre-release supplement to increase efficacy of SIT programs. Coffee powder and guarana powder both contain caffeine but were not effective in promoting sexual maturation. Q-fly SIT programs commonly release immature flies and pre-release caffeine supplements provide a new means to decrease the delay to maturation following release and thereby increase the number of flies that survive to maturity and participate in mating with pest populations. Having proven effective for Q-fly, it is highly likely that caffeine supplements will yield parallel benefits in other fruit flies.

## Methods

### Study insects

Q-flies were obtained as pupae from the Fruit Fly Production Facility at the Elizabeth Macarthur Agricultural Institute, Menangle, New South Wales (NSW), Australia (for production details, see Dominiak et al.^86^). At this facility, larvae were reared on lucerne chaff larval diet. At Macquarie University, all pupae and flies, and experiments, were maintained in controlled environment rooms at 25 ± 0.5 °C and 65 ± 5% RH. A L13:D11 h photoperiod was maintained, with flies experiencing a simulated dawn and dusk as the lights ramped up and down through 0.5 h at the beginning and end of the light phase, respectively. To sterilize flies, pupae were irradiated with 65 Gy gamma radiation at Macquarie University, New South Wales, Australia^87,88^. For sterilisation, ca. 4000 pupae were sealed in zip lock plastic bags (100 × 150 mm) two days prior to emergence. The sealed bags were held overnight in a temperature-controlled room (18 °C) to so that they were hypoxic for irradiation.

### Treatments

Newly emerged adult male Q-flies were provided dry blended mixtures of the following supplements for 48 h:

- Analytical caffeine: Analytical caffeine + sugar and YH (3:1).
- Guarana powder: Guarana powder + sugar and YH (3:1).
- Instant coffee: Instant coffee + sugar and YH (3:1).
- Control: Only sugar and YH (3:1) as food.

All the caffeine sources used in this study were available in powder form. The powdered caffeine source (as required on dry weight basis) was mixed with sugar and YH (3:1) using a blender such that selective feeding was not possible.

### Analysis of caffeine contents

Analytical caffeine was obtained from Sigma Aldrich Australia (CAS Number 58-08-2), Guarana powder was from My Protein Australia Ltd, and instant coffee was from Moconna classic medium roast (Netherlands). To estimate caffeine content of guarana powder and instant coffee, GC-FID analysis was performed on a Shimadzu GC17A equipped with a split/split less injector, a Restek Rxi-5Ms fused silica capillary column (30 m × 0.25 mm, 0.25 μm film), flame ionization detector (FID) and an AOC-20 auto sampler. Hydrogen gas (BOC, North Ryde, NSW, Australia) (99.999%) was used as a carrier gas with a constant flow of 1.5 mL/min. Caffeine in guarana and instant coffee was extracted by dissolving in boiling water and extracting with dichloromethane (DCM). A weighed amount of guarana powder or instant coffee was added to water (15 mL) in a beaker. The solution was boiled and stirred with magnetic stirrer bar for 15 min. The solution was vacuum filtered, and the filtrates were allowed to cool to room temperature. The filtrate was extracted with DCM (15 mL × 3) using a separator funnel. The organic layers were combined, dried over anhydrous Na_2_SO_4_ (Sigma Aldrich), and concentrated under reduced pressure to give 1.0 mL, which was subjected to GC-FID for quantification. A stock solution of caffeine was prepared. Standard solutions of caffeine were prepared by serial dilution of the stock solution. Tridecane (Sigma Aldrich) was used as an internal standard that was incorporated into each standard and sample solutions to give a final concentration of 8.53 µg/mL. The standard and sample solutions were run through GC-FID. The peak area ratios of GC response of caffeine to internal standard in standard solutions were plotted against the concentrations of the standard solutions to obtain a standard curve. The standard curve was used to estimate the concentration of caffeine and hence the amount in each sample could be calculated in µg/g. Guarana powder and instant coffee used in the analysis contained 5% and 1% caffeine, respectively.

### Longevity

Approximately 6,000 pupae from a single day of pupation were placed in each of 22 mesh cages (Megaview Bugdorm 44,545, 47.5 × 47.5 × 47.5 cm) for adult emergence. Usually few flies emerge on the first day of emergence, and these were discarded. During emergence, each cage of flies was provided with water-soaked cotton wool in two 70-mL clear plastic sample container but no food. After the second day of emergence, unemerged pupae were removed from the cages and newly emerged adults (age 0–24 h) were provided a 1:3 mixture of yeast hydrolysate and sugar containing one of seven doses of analytical caffeine (0.0125, 0.025, 0.05, 0.075, 0.1, 0.125, & 0.15%), guarana powder (0.25, 0.50, 1, 1.5, 2 , 2.5, & 3%), instant coffee (1.25, 2.5, 5, 7.5, 10, 12.5, & 15%) or a control (0%) for 2 days ad libitum. Based on caffeine quantification, doses from the three sources had equivalent caffeine levels. After two days, five male flies from each treatment were placed in each of five 1.125 L cages that had a mesh-covered window (ca. 28 cm^2^) for ventilation (i.e., 25 flies for each treatment). Each cage was provided with water-soaked cotton wool and sugar in separate 35 mm Petri dishes. Flies were checked daily until all had died. Dead flies were removed from the cages daily. Longevity trials were performed twice, using pupae from two different batches.

### Sexual performance

To assess the effect of caffeine on mating propensity, the two highest doses that were found to be non-toxic for all caffeine sources in the longevity assay were tested. Approximately 6,000 pupae from a single day of pupation were placed in each of seven mesh cages (Megaview Bugdorm 44,545 Taiwan) for adult emergence. Each cage was provided with water-soaked cotton wool in two 70-mL clear plastic sample containers during emergence^26,27^. Flies emerging on the first day of emergence were discarded^26,27^. After the second day of emergence, newly emerged adults (age 0–24 h) were provided 1:3 mixture of yeast hydrolysate and sugar containing analytical caffeine (0.075 or 0.1%), guarana powder (1.5 or 2%), instant coffee (7.5 or 10%) or a control (0%) for next two days. Doses of analytical caffeine, guarana powder and instant coffee had equivalent caffeine content. After two days, the treated food was replaced with sugar only in a 90 mm Petri dish^27^. The flies were then sorted according to sex within 3 days after emerging by collecting and transferring individual flies in glass tubes to transparent plastic 12-L cages that had a mesh-covered ca. 80 cm^2^ window for ventilation^26,27^. Approximately 200 flies in each 12-L cage were provided continuous access to dry granular sucrose^27^. As a source of water, cages were supplied with water-soaked cotton wool in a 70-mL clear plastic sample container^27^.

To obtain mature flies (12–17 days old) to pair with treated flies, ca. 800 pupae from numerous pupation dates were placed in separate mesh cages (Megaview Bugdorm 44,545) for adult emergence. Cages were supplied with water-soaked cotton wool two 70-mL sample containers and dry granular sucrose along with yeast hydrolysate (3:1) as food on a 90 mm Petri dish; this diet is effective at supporting Q-fly development^20–22^. Adult flies were sorted according to sex within 3 days after emerging by collecting and transferring individual flies in glass tubes to clear plastic 12-L cages that had a mesh-covered ca. 80 cm^2^ window for ventilation^26,27^. Approximately 200 flies in each 12-L cage were provided continuous access to dry blended mixture of granular sucrose and yeast hydrolysate (3:1). Cages were supplied with water-soaked cotton wool in a 70-mL clear plastic sample container. No calling, courting, or mating was observed in cages prior to separating the sexes.

Mating trials were conducted at 4, 6, 8, 10, 12, 15, and 20 days post emergence. Mating in Q-flies takes place at dusk^89^. On each mating day, at least four hours before the onset of dusk, twenty males from each treatment group were placed individually in clear plastic 1.125 L containers that had a mesh-covered window (ca. 28 cm^2^) for ventilation^27^. Each male fly was paired with a sexually mature virgin (12–17 days old) female fly^20^. In case of early matings, periodic observations were carried out every 15–20 min from when pairs were set up, and continuous observations in which each cage was observed at least once each minute began 90 min prior to the onset of dusk^27^. To assess copula latency, the time of onset of copulation was recorded for each mating pair^27,28^. To assess copula duration for each mating pair, observations continued until the last pair had separated^27,28^. Overall, 140 male flies were tested on each day, providing a total of 980 test pairs across all ages. The experiment was performed twice using batches of pupae obtained two months apart.

### Reproductive organ development

Application of treatments for assessment of reproductive development was similar to that for assessment of mating propensity (above). To assess the effect of caffeine on male reproductive organ development, we measured area and length of testes and ejaculatory apodeme. Ten males were collected from each treatment at 4, 6, 8, 10, 12, 15, and 20 days post emergence and were dissected in phosphate-buffered saline (PBS; pH 7.4) using fine forceps on a microscope slide under a Leica MZ6 stereomicroscope. Then the dissected ejaculatory apodeme and testes were photographed using a 1.3-megapixel camera (Model-AM4023CT C-Mount camera; Dino-Lite digital microscope, Taiwan) through the phototube of the stereomicroscope. Images were calibrated and measured using Image J (Version1.49, NIH, Maryland, USA). Ejaculatory apodeme length was measured from where the ejaculatory sac joins the apodeme to the farthest point, and area was measured by tracing the outline of the apodeme following Radhakrishnan and Taylor^75^. Length of the testes was measured by tracing a midline through the centre of the organ from the base to the curved tip, and the area of testes was measured by tracing the outline^75^. The experiment was performed twice using batches of pupae obtained 2 months apart.

### Statistical analysis

All analyses were conducted using R v3.5.1. Survival of the flies (log-transformed) was assessed using a general linear mixed model (GLMM). Data were not censored and followed a Gaussian distribution (based on model residuals). Separate models were considered for each caffeine supplement with each having control as dose of zero. Dose was included as a categorical variable due to a sharp drop off in survival in two treatments. Replicate was included as a fixed effect and cage identity was included as a random effect. Post-hoc comparisons were then performed comparing the control to each dose using Dunnett’s correction.

Mating probability (binary outcome) was assessed using a general linear model with a binomial distribution. For both mating latency and mating duration (square-root transformed) a linear model (Gaussian distribution) was used. Latency was re-defined as the time from 30 min post-dusk as the square-root of this variable was normally distributed, but all latency results are back-transformed to the original definition (time to initiate mating). Separate models were run for each caffeine supplement. However, the same control flies were included in each model and defined as a dose of zero. The predictors were the same for each model: dose (ordinal), age (ordinal), and replicate (nominal). Post-hoc comparisons for each dose compared with the control were then performed using least-square means with Dunnett’s correction.

Area and length (both log-transformed) of ejaculatory apodeme and testis were analysed in separate general linear models (GLM, Gaussian distribution) for each caffeine supplement. As above, the control was included as dose of zero for each treatment. Replicate was also included as fixed effect. Post-hoc comparisons for each dose compared with the control were then performed using least-square means with Dunnett’s correction.

## Data Availability

The datasets generated during and/or analysed during the current study are available from the corresponding author on reasonable request.

## References

[1] Qin Y, Paini DR, Wang C, Fang Y, Li Z (2015). Global establishment risk of economically important fruit fly species (Tephritidae). PLoS ONE.

[2] White IM, Elson-Harris MM (1992). Fruit Flies of Economic Significance: Their Identification and Bionomics.

[3] Clarke AR, Powell KS, Weldon CW, Taylor PW (2011). The ecology of *Bactrocera tryoni* (Diptera: Tephritidae): What do we know to assist pest management?. Ann. Appl. Biol..

[4] Dominiak BC, Daniels D (2012). Review of the past and present distribution of Mediterranean fruit fly (*Ceratitis capitata* Wiedemann) and Queensland fruit fly (*Bactrocera tryoni* Froggatt) in Australia. Aust. J. Entomol..

[5] Hancock, D., Hamacek, E., Lloyd, A. & Elson-Harris, M. The distribution and host plants of fruit flies (Diptera: Tephritidae) in Australia. Department of Primary Industries, Queensland, Australia. Information Series Q 199067, 1–75 (2000).

[6] Benelli G (2014). Sexual communication and related behaviours in Tephritidae: Current knowledge and potential applications for Integrated Pest Management. J. Pest Sci..

[7] Knipling E (1955). Possibilities of insect control or eradication through the use of sexually sterile males. J. Econ. Entomol..

[8] Reyes J, Vreyson MJB, Robinson AS, Hendrichs J (2007). A multi-institutional approach to create fruit fly-low prevalence and fly-free areas in Central America. Area-Wide Control of Insect Pests: From Research to Field Implementation.

[9] Kakinohana H, Calkins CO, Klassen W, Liedo P (1994). The melon fly eradication program in Japan. Fruit Flies and the Sterile Insect Technique.

[10] Yosiaki I, Kakinohana H, Yamagishi M, Kohama T (2003). Eradication of the melon fly, *Bactrocera cucurbitae*, from Okinawa, Japan, by means of the sterile insect technique, with special emphasis on the role of basic studies. J. Asia Pac. Entomol..

[11] Orankanok W, Chinvinijkul S, Thanaphum S, Sitilob P, Enkerlin W, Vreyson MJB, Robinson AS, Hendrichs J (2007). Area-wide integrated control of oriental fruit fly *Bactrocera dorsalis* and guava fruit fly *Bactrocera correcta* in Thailand. Area-Wide Control of Insect Pests: From Research to Field Implementation.

[12] Orozco-Dávila D, de Lourdes Adriano-Anaya M, Quintero-Fong L, Salvador-Figueroa M (2015). Sterility and sexual competitiveness of Tapachula-7 *Anastrepha ludens* males irradiated at different doses. PLoS ONE.

[13] Liedo P, De Leon E, Barrios M, Valle-Mora J, Ibarra G (2002). Effect of age on the mating propensity of the Mediterranean fruit fly (Diptera: Tephritidae). Fla. Entomol..

[14] Pérez-Staples D, Harmer AM, Collins SR, Taylor PW (2008). Potential for pre-release diet supplements to increase the sexual performance and longevity of male Queensland fruit flies. Agric. Forest Entomol..

[15] Reynolds, O. *et al.* Enhancing emergence and release methods of the sterile insect technique (SIT) to improve market access. Report to Horticulture Australia Limited MT06049. Horticultural Australia Ltd., Sydney (2012).

[16] Weldon CW, Pérez-Staples D, Taylor PW (2008). Feeding on yeast hydrolysate enhances attraction to cue-lure in Queensland fruit flies, *Bactrocera tryoni*. Entomol. Exp. Appl..

[17] Drew RA, Yuval B, Aluja M, Norrbom AL (2000). The evolution of fruit fly feeding behavior. Fruit Flies (Tephritidae) Phylogeny and Evolution of Behavior.

[18] Fanson BG, Taylor PW (2012). Additive and interactive effects of nutrient classes on longevity, reproduction, and diet consumption in the Queensland fruit fly (*Bactrocera tryoni*). J. Insect Physiol..

[19] Meats A, Leighton S (2004). Protein consumption by mated, unmated, sterile and fertile adults of the Queensland fruit fly, *Bactrocera tryoni* and its relation to egg production. Physiol. Entomol..

[20] Pérez-Staples D, Prabhu V, Taylor PW (2007). Post-teneral protein feeding enhances sexual performance of Queensland fruit flies. Physiol. Entomol..

[21] Vijaysegaran S, Walter G, Drew R (2002). Influence of adult diet on the development of the reproductive system and mating ability of Queensland fruit fly *Bactrocera tryoni* (Frogratt)(Diptera: Tephritidae). J. Trop. Agric. Food Sci..

[22] Pérez-Staples D, Weldon CW, Taylor PW (2011). Sex differences in developmental response to yeast hydrolysate supplements in adult Queensland fruit fly. Entomol. Exp. Appl..

[23] Weldon CW, Taylor PW (2011). Sexual development of wild and mass-reared male Queensland fruit flies in response to natural food sources. Entomol. Exp. Appl..

[24] Reynolds O, Orchard B, Collins S, Taylor PW (2014). Yeast hydrolysate supplementation increases field abundance and persistence of sexually mature sterile Queensland fruit fly, *Bactrocera tryoni* (Froggatt). Bull. Entomol. Res..

[25] Adnan SM, Farhana I, Inskeep JR, Rempoulakis P, Taylor PW (2020). Dietary methoprene enhances sexual competitiveness of sterile male Queensland fruit flies in field cages. J. Pest Sci..

[26] Adnan SM, Farhana I, Inskeep JR, Rempoulakis P, Taylor PW (2019). Accelerated Sexual maturation in methoprene-treated sterile and fertile male Queensland fruit flies (Diptera: Tephritidae), and mosquito larvicide as an economical and effective source of methoprene. J. Econ. Entomol..

[27] Adnan SM (2018). Dietary methoprene supplement promotes early sexual maturation of male Queensland fruit fly *Bactrocera tryoni*. J. Pest Sci..

[28] Collins SR, Reynolds OL, Taylor PW (2014). Combined effects of dietary yeast supplementation and methoprene treatment on sexual maturation of Queensland fruit fly. J. Insect Physiol..

[29] Haq I (2010). Methoprene modulates the effect of diet on male melon fly, *Bactrocera cucurbitae*, performance at mating aggregations. Entomol. Exp. Appl..

[30] Haq I (2010). Effects of the juvenile hormone analogue methoprene and dietary protein on male melon fly *Bactrocera cucurbitae* (Diptera: Tephritidae) mating success. J. Insect Physiol..

[31] Abraham S (2013). Remating behavior in *Anastrepha fraterculus* (Diptera: Tephritidae) females is affected by male juvenile hormone analog treatment but not by male sterilization. Bull. Entomol. Res..

[32] Liendo MC (2013). Precocious sexual signalling and mating in *Anastrepha fraterculus* (Diptera: Tephritidae) sterile males achieved through juvenile hormone treatment and protein supplements. Bull. Entomol. Res..

[33] Segura D (2013). Methoprene treatment reduces the pre-copulatory period in *Anastrepha fraterculus* (Diptera: Tephritidae) sterile males. J. Appl. Entomol..

[34] Segura DF (2009). Enhancing mating performance after juvenile hormone treatment in *Anastrepha fraterculus*: A differential response in males and females acts as a physiological sexing system. Entomol. Exp. Appl..

[35] Gómez Y, Teal P, Pereira R (2013). Enhancing efficacy of Mexican fruit fly SIT programmes by large-scale incorporation of methoprene into pre-release diet. J. Appl. Entomol..

[36] Gomez-Simuta Y, Diaz-Fleisher F, Arredondo J, Díaz-Santiz E, Pérez-Staples D (2017). Precocious Mexican fruit fly methoprene-fed males inhibit female receptivity and perform sexually as mature males. J. Appl. Entomol..

[37] Pereira R, Teal P, Conway H, Worley J, Sivinski J (2013). Influence of methoprene and dietary protein on maturation and sexual performance of sterile *Anastrepha ludens* (Diptera: Tephritidae). J. Appl. Entomol..

[38] Pereira R, Sivinski J, Teal PE (2009). Influence of methoprene and dietary protein on male *Anastrepha suspensa* (Diptera: Tephritidae) mating aggregations. J. Insect Physiol..

[39] Pereira R, Sivinski J, Teal PE (2010). Influence of a juvenile hormone analog and dietary protein on male *Anastrepha suspensa* (Diptera: Tephritidae) sexual success. J. Econ. Entomol..

[40] Haq I, Vreysen MJ, Cacéres C, Shelly TE, Hendrichs J (2015). Optimizing methyl-eugenol aromatherapy to maximize posttreatment effects to enhance mating competitiveness of male *Bactrocera carambolae* (Diptera: Tephritidae). Insect Sci..

[41] Ji Q, Chen J, McInnis D, Guo Q (2013). The effect of methyl eugenol exposure on subsequent mating performance of sterile males of *Bactrocera dorsalis*. J. Appl. Entomol..

[42] Orankanok W, Chinvinijkul S, Sawatwangkhoung A, Pinkaew S, Orankanok S (2013). Methyl eugenol and pre-release diet improve mating performance of young Bactrocera dorsalis and *Bactrocera correcta* males. J. Appl. Entomol..

[43] Shelly T, Rendon P, Moscoso F, Menendez R (2010). Testing the efficacy of aromatherapy at the world’s largest eclosion facility for sterile males of the Mediterranean fruit fly (Diptera: Tephritidae). Proc. Hawaiian Entomol. Soc..

[44] Shelly TE, Edu J (2008). Do methyl eugenol-fed males of the oriental fruit fly (Diptera: Tephritidae) induce female re-mating?. Fla. Entomol..

[45] Shelly TE, Edu J, McInnis D (2010). Pre-release consumption of methyl eugenol increases the mating competitiveness of sterile males of the oriental fruit fly, *Bactrocera dorsalis*, in large field enclosures. J. Insect Sci..

[46] Shelly TE, Edu J, Pahio E (2005). Influence of diet and methyl eugenol on the mating success of males of the oriental fruit fly, *Bactrocera dorsalis* (Diptera: Tephritidae). Fla. Entomol..

[47] Haq I, Cáceres C, Meza JS, Hendrichs J, Vreysen MJ (2018). Different methods of methyl eugenol application enhance the mating success of male Oriental fruit fly (Dipera: Tephritidae). Sci. Rep..

[48] Kumaran N, Prentis PJ, Mangalam KP, Schutze MK, Clarke AR (2014). Sexual selection in true fruit flies (Diptera: Tephritidae): Transcriptome and experimental evidences for phytochemicals increasing male competitive ability. Mol. Ecol..

[49] Akter H, Mendez V, Morelli R, Pérez J, Taylor PW (2017). Raspberry ketone supplement promotes early sexual maturation in male Queensland fruit fly, *Bactrocera tryoni* (Diptera: Tephritidae). Pest Manag. Sci..

[50] Alhaider IA, Aleisa AM, Tran TT, Alzoubi KH, Alkadhi KA (2010). Chronic caffeine treatment prevents sleep deprivation-induced impairment of cognitive function and synaptic plasticity. Sleep.

[51] Andretic R, Kim YC, Jones FS, Han KA, Greenspan RJ (2008). Drosophila D1 dopamine receptor mediates caffeine-induced arousal. Proc. Natl. Acad. Sci. USA.

[52] Daly J, Garattini S (1993). Mechanism of action of caffeine. Caffeine, Coffee, and Health.

[53] Nall AH (2016). Caffeine promotes wakefulness via dopamine signaling in Drosophila. Sci. Rep..

[54] Nishi Y, Sasaki K, Miyatake T (2010). Biogenic amines, caffeine and tonic immobility in *Tribolium castaneum*. J. Insect Physiol..

[55] Penetar D (1993). Caffeine reversal of sleep deprivation effects on alertness and mood. Psychopharmacology.

[56] Roehrs T, Roth T (2008). Caffeine: Sleep and daytime sleepiness. Sleep Med. Rev..

[57] Arita LH, Kaneshiro KY (1988). Body size and differential mating success between males of two populations of the Mediterranean fruit fly. Pac. Sci..

[58] Aquino JCD, Souza CFC, Santos JRDJ, Joachim-Bravo IS (2016). Adding guarana powder to medfly diets: An alternative for improving the Sterile Insect Technique. Sci. Agric..

[59] Kucharski R, Maleszka R (2002). Evaluation of differential gene expression during behavioral development in the honeybee using microarrays and northern blots. Genome Biol..

[60] Cruz D (2016). Caffeine impacts in the clam *Ruditapes philippinarum*: Alterations on energy reserves, metabolic activity and oxidative stress biomarkers. Chemosphere.

[61] Nehlig A, Gupta BS, Gupta U (1999). Cerebral energy metabolism and blood flow: useful tools for the understanding of the behavioral effects of caffeine. Caffeine and Behavior, Current Views and Research Trends.

[62] Kume K, Kume S, Park SK, Hirsh J, Jackson FR (2005). Dopamine is a regulator of arousal in the fruit fly. J. Neurosci..

[63] Meehan MJ, Wilson R (1987). Locomotor activity in theTyr-1 mutant of *Drosophila melanogaster*. Behav. Genet..

[64] Pendleton RG, Rasheed A, Hillman R (2000). Effects of adrenergic agents on locomotor behavior and reproductive development in Drosophila. Drug Dev. Res..

[65] Pendleton RG, Rasheed A, Sardina T, Tully T, Hillman R (2002). Effects of tyrosine hydroxylase mutants on locomotor activity in Drosophila: A study in functional genomics. Behav. Genet..

[66] Wicker-Thomas C, Hamann M (2008). Interaction of dopamine, female pheromones, locomotion and sex behavior in *Drosophila melanogaster*. J. Insect Physiol..

[67] Adamo S, Linn C, Hoy R (1995). The role of neurohormonal octopamine during fight or flight behaviour in the field cricket *Gryllus bimaculatus*. J. Exp. Biol..

[68] Stevenson PA, Hofmann HA, Schoch K, Schildberger K (2000). The fight and flight responses of crickets depleted of biogenic amines. J. Neurobiol..

[69] Božič J, Woodring J (1998). Variations of brain biogenic amines in mature honeybees and induction of recruitment behavior. Comp. Biochem. Physiol. A Mol. Integr. Physiol..

[70] Menzel R, Heyne A, Kinzel C, Gerber B, Fiala A (1999). Pharmacological dissociation between the reinforcing, sensitizing, and response-releasing functions of reward in honeybee classical conditioning. Behav. Neurosci..

[71] Harano KI, Sasaki M, Nagao T, Sasaki K (2008). Dopamine influences locomotor activity in honeybee queens: Implications for a behavioural change after mating. Physiol. Entomol..

[72] Harano KI, Sasaki K, Nagao T (2005). Depression of brain dopamine and its metabolite after mating in European honeybee (*Apis mellifera*) queens. Naturwissenschaften.

[73] Casagrand JL, Ritzmann RE (1992). Biogenic amines modulate synaptic transmission between identified giant interneurons and thoracic interneurons in the escape system of the cockroach. J. Neurobiol..

[74] Goldstein RS, Camhi JM (1991). Different effects of the biogenic amines dopamine, serotonin and octopamine on the thoracic and abdominal portions of the escape circuit in the cockroach. J. Comp. Physiol. A.

[75] Radhakrishnan P, Taylor PW (2008). Ability of male Queensland fruit flies to inhibit receptivity in multiple mates, and the associated recovery of accessory glands. J. Insect Physiol..

[76] Collins SR, Pérez-Staples D, Taylor PW (2012). A role for copula duration in fertility of Queensland fruit fly females mated by irradiated and unirradiated males. J. Insect Physiol..

[77] Harmer AM, Radhakrishnan P, Taylor PW (2006). Remating inhibition in female Queensland fruit flies: Effects and correlates of sperm storage. J. Insect Physiol..

[78] Belitz HD, Grosch W, Schieberle P, Belitz HD, Grosch W, Schieberle P (2009). Coffee, tea, cocoa. Food Chemistry.

[79] Trugo L, Caballero B, Trugo L, Finglas PM (2003). Coffee. Encyclopedia of Food Sciences and Nutrition.

[80] Lima D (2003). Café e Saúde: Manual de farmacologia clinica, terapeutica e toxicologia. Rio de Janeiro MEDSI.

[81] Heckman MA, Weil J, De Mejia EG (2010). Caffeine (1,3,7-trimethylxanthine) in foods: A comprehensive review on consumption, functionality, safety, and regulatory matters. J. Food Sci..

[82] Basile A (2005). Antibacterial and antioxidant activities of ethanol extract from *Paullinia cupana* Mart. J. Ethnopharmacol..

[83] Marx F (1990). Analysis of guarana seeds II. Studies on the composition of the tannin fraction. Zeitschrift für Lebensmittel-untersuchung und Forschung.

[84] Ushirobira TMA (2007). Chemical and microbiological study of extract from seeds of guaraná (*Paullinia cupana* var. sorbilis). Acta Farm Bonaerense.

[85] Yamaguti-Sasaki E (2007). Antioxidant capacity and in vitro prevention of dental plaque formation by extracts and condensed tannins of *Paullinia cupana*. Molecules.

[86] Dominiak BC, Sundaralingam S, Jiang L, Jessup A, Barchia I (2008). Production levels and life history traits of mass reared Queensland fruit fly *Bactrocera tryoni* (Froggatt) (Diptera: Tephritidae) during 1999/2002 in Australia. Plant Prot. Q..

[87] Collins S, Weldon CW, Banos C, Taylor PW (2009). Optimizing irradiation dose for sterility induction and quality of *Bactrocera tryoni*. J. Econ. Entomol..

[88] Dominiak B (2014). Evaluating irradiation dose for sterility induction and quality control of mass-produced fruit fly *Bactrocera tryoni* (Diptera: Tephritidae). J. Econ. Entomol..

[89] Tychsen PH, Fletcher BS (1971). Studies on the rhythm of mating in the Queensland fruit fly, *Dacus tryoni*. J. Insect Physiol..

